# Managing multiple sclerosis in individuals aged 55 and above: a comprehensive review

**DOI:** 10.3389/fimmu.2024.1379538

**Published:** 2024-04-05

**Authors:** Óscar Fernández, Per Soelberg Sörensen, Giancarlo Comi, Patrick Vermersch, Hans-Peter Hartung, Letizia Leocani, Thomas Berger, Bart Van Wijmeersch, Celia Oreja-Guevara

**Affiliations:** ^1^ Departament of Pharmacology, Faculty of Medicine; Institute of Biomedical Research of Malaga (IBIMA), Regional University Hospital of Malaga, Malaga, Spain; ^2^ Department of Pharmacology and Pediatry, Faculty of Medicine, University of Malaga, Malaga, Spain; ^3^ Danish Multiple Sclerosis Center, Department of Neurology, Copenhagen University Hospital, Rigshospitalet, Copenhagen, Denmark; ^4^ Copenhagen and Department of Clinical Medicine, Faculty of Health and Medical Sciences, University of Copenhagen, Copenhagen, Denmark; ^5^ Department of Neurorehabilitation Sciences, Multiple Sclerosis Centre Casa di Cura Igea, Milan, Italy; ^6^ University Vita-Salute San Raffaele, Milan, Italy; ^7^ Univ. Lille, Inserm U1172 LilNCog, CHU Lille, FHU Precise, Lille, France; ^8^ Department of Neurology, Medical Faculty, Heinrich-Heine-University, Düsseldorf, Germany; ^9^ Brain and Mind Center, University of Sydney, Sydney, NSW, Australia; ^10^ Department of Neurology, Palacky University Olomouc, Olomouc, Czechia; ^11^ Department of Neurology, Medical University of Vienna, Vienna, Austria; ^12^ Comprehensive Center for Clinical Neurosciences & Mental Health, Medical University of Vienna, Vienna, Austria; ^13^ University MS Centre, Hasselt-Pelt, Belgium; ^14^ Rehabilitation and Multiple Sclerosis (MS), Noorderhart Hospitals, Pelt, Belgium; ^15^ Department of Neurology, Hospital Clínico Universitario San Carlos, IdISSC, Madrid, Spain; ^16^ Department of Medicine, Faculty of Medicine, Complutense University of Madrid, Madrid, Spain

**Keywords:** multiple sclerosis, aging, management, disease-modifying treatments, symptomatic treatment

## Abstract

Multiple Sclerosis (MS) management in individuals aged 55 and above presents unique challenges due to the complex interaction between aging, comorbidities, immunosenescence, and MS pathophysiology. This comprehensive review explores the evolving landscape of MS in older adults, including the increased incidence and prevalence of MS in this age group, the shift in disease phenotypes from relapsing-remitting to progressive forms, and the presence of multimorbidity and polypharmacy. We aim to provide an updated review of the available evidence of disease-modifying treatments (DMTs) in older patients, including the efficacy and safety of existing therapies, emerging treatments such as Bruton tyrosine kinase (BTKs) inhibitors and those targeting remyelination and neuroprotection, and the critical decisions surrounding the initiation, de-escalation, and discontinuation of DMTs. Non-pharmacologic approaches, including physical therapy, neuromodulation therapies, cognitive rehabilitation, and psychotherapy, are also examined for their role in holistic care. The importance of MS Care Units and advance care planning are explored as a cornerstone in providing patient-centric care, ensuring alignment with patient preferences in the disease trajectory. Finally, the review emphasizes the need for personalized management and continuous monitoring of MS patients, alongside advocating for inclusive study designs in clinical research to improve the management of this growing patient demographic.

## 1 Introduction

Multiple Sclerosis (MS) is a chronic autoimmune, neurodegenerative disease characterized by inflammation, demyelination and axonal degeneration in the central nervous system (CNS). The number of people affected by the disease is estimated to be 2.9 million worldwide (1–3). Historically, MS has mainly affected younger adults, with first symptoms often presenting between the ages of 20 and 40. However, a growing body of literature underscores an evolving epidemiology with an increasing incidence and prevalence in older individuals (4–6). This changing demographic landscape not only offers an opportunity to understand the disease intricacies within an older population but also mandates an in-depth examination of existing and potential management strategies. In this article, we provide a succinct overview of the epidemiology, symptomatology, and pathophysiological events, and a deeper review of the use of disease-modifying treatment (DMTs) in older MS patients, including data from randomized clinical trials (RCT) and real-world evidence (RWE), and pharmacologic and non-pharmacologic symptomatic treatment, including reference to the important role of MS care units (MSCU). The article concludes by discussing the strategies for planning care in the advanced age and offers some practical insights for neurologists.

A key consideration is the distinction between chronological age (the number of years a person has lived) and biological age (aging-driven biological changes, such as molecular and cellular degradation). In this article, our focus will be on chronological age, as this is the metric employed in the vast majority of clinical studies evaluating management of MS patients.

### 1.1 Epidemiology: incidence and prevalence of MS in older adults

MS can no longer be regarded as a condition of early adulthood. Several studies have documented an increase in the prevalence of MS among older adults in the recent years (3–5, 7–9). For instance, a study in Canada showed that the peak prevalence moved from 50-54 years in the eighties to 55-59 years in the 2000s (5). Similarly, in the US, the highest age-specific prevalence of MS in 2010 was observed in the 55-64 age group, followed by the 65-74 range (7). Similar results were found in Denmark, where the prevalence peaked at ages 55-59 years for women and 60-64 years for men in 2013 (9). This rise in the prevalence of MS in older adults might be linked to the fact that life expectancy of people living with MS has progressively increased in the last years (10, 11), even if it is still 6-10 years less than in the general population (10, 12, 13). Factors contributing to the extended life expectancy of patients with MS might include early initiation of therapy (14), the introduction of high-efficacy DMTs (heDMTs) and their earlier use (15, 16), advancements in symptomatic treatments (17), the establishment of multidisciplinary care models (18), and lifestyle interventions (19, 20).

Moreover, there is evidence that the age of onset of MS has also shifted forward over the last five decades (21, 22). When the onset of MS occurs at the age of 50 years or older, it is referred to as late-onset MS (LOMS), and when it occurs after 60 it is known as very-late-onset MS (VLOMS). A rise in the incidence of LOMS has also been observed in European countries (3, 22). A study in Italy showed that the percentage of individuals diagnosed with LOMS rose from 1% prior to 1991 to nearly 10% after 2010 (22). The rise in incidence of LOMS might be due to more awareness with this population and improvement of diagnostic tools. However, it does not explain why the increase in incidence in older individuals is seen particularly in women. Moreover, in LOMS and VLOMS, the time to diagnosis is almost double that of adult early-onset MS (AEOMS), indicating notably longer delays in diagnosis for older patients (23).

**Figure 1:**
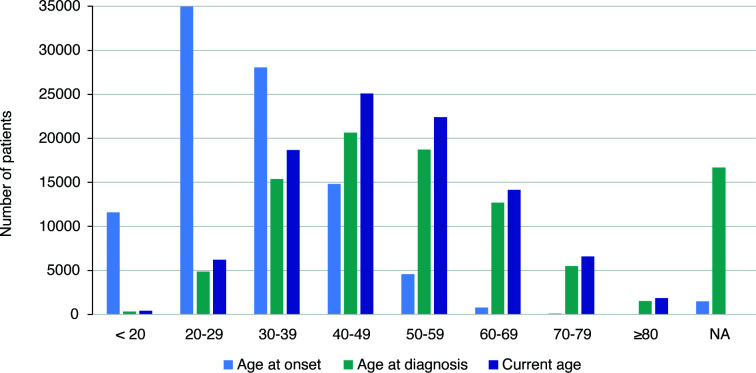
Age distribution of patients included in the MSBase registry.

The rise of prevalence of MS in older patients poses a great burden not only to those affected but also to society as a whole. This concern is particularly pertinent given that the highest levels of life-years lost due to premature death or disability occur predominantly in the sixth decade of life (24). **Figure 1** illustrated the age at onset, age at diagnosis, and current age of patients included in the MSBase registry, a renowned international online registry that collects longitudinal, real-world data from people with MS mainly in MS centers. The registry has recorded data of 96,352 patients so far (25), providing updated complementary information of the age distribution of MS.

### 1.2 Symptomatology and quality of life

The symptoms already present at a young age —spasticity, fatigue, pain, bladder, bowel and sexual dysfunction, dizziness, vertigo, balance issues, sleep and cognitive impairments, and emotional dysregulation — (17, 26–28) persist and often worsen as patients with MS age. In older patients some of these symptoms may be underrecognized or overlooked owing to the presence of comorbidities (28, 29). While some symptoms of MS, such as impairments in walking and bladder function continuously worsen throughout the age spectrum (30, 31), anxiety or depression might remain stable or even decrease with age (30–36). MS symptoms and age symptoms can overlap, complicating the diagnosis and management of MS in the elderly. For example, micturition alterations, is a typical symptom of MS but can also arise from prostate disease, a common condition in older men.

In the case of LOMS, the predominant symptoms encompass motor dysfunctions, sensory disturbances, visual impairments, and cerebellar symptoms (23, 37–39). These patients frequently exhibit a primary progressive phenotype, and, when they present a relapsing-remitting MS (RRMS) phenotype, an earlier conversion to secondary progressive MS (SPMS) compared to those diagnosed at a younger age is observed (37, 38, 40, 41). Onset at age 50 indeed tripled risks of developing SPMS compared to onset at age 20 (40). For equivalent disease duration, LOMS has been associated with higher Expanded Disability Status Scale (EDSS) (41). A study including 3,352 patients with age at onset <50 years and 245 patients with age at onset ≥50 years showed that, once EDSS 4 is achieved, age at disease onset was the only predictor of progression of irreversible disability (42).

The age-related decline in physical health in patients with MS has been estimated to be accelerated by 15–30 years compared to healthy old individuals (43). Pre-existing disability and older age have been identified as the principal risk factors for further disability accumulation (44). The gradual loss of mobility and independence inevitably affect the health-related quality of life (HRQoL) of patients and their families (45–47). Disability, fatigue, pain, spasms, stiffness, depression, and cognitive impairment have been pinpointed as risk factors for poor HRQoL in MS (46, 48, 49), while higher heightened self-esteem, self-efficacy, resilience and social support have demonstrated to be protective (46). In older adults with MS, in particular, physical HRQoL was positively associated with the patient being employed and widowed (likely because they now assume responsibilities previously shared with their spouse), whereas mental HRQoL was negatively associated with a lower level of education (50).

### 1.3 Underlying pathophysiology

MS manifests differently throughout the lifespan, with inflammation and neurodegeneration being two pivotal processes that shape the course of the disease. In older adults, the patterns of inflammation and neurodegeneration often intertwine differently than in younger populations. The initial phase of MS is dominated by waves of inflammation driven by peripheral immune mechanisms, with relapses and magnetic resonance imaging (MRI) lesions and pathologically areas of demyelination and variable axonal loss which can be already observed in radiologically isolated syndrome (RIS) (51) However, with the increase of disease duration and age, relapse frequency and new inflammatory MRI lesions decline (52–55) and the disease often transitions from the relapsing-remitting phase to the progressive phase. This progressive phase is marked by a compartmentalized inflammation due to a combination of variable neurodegenerative mechanisms with a key role of smoldering plaques, characterized by microglial activation and slow expansion of pre-existing plaques (44, 54, 56–58). In a study that examined changes in immune cell composition and activation levels in the CSF related to age in patients with RRMS and primary progressive (PPMS) an age-dependent decrease in counts of B and T cells, plasma cells and natural killer cells was noted in patients with PPMS, but not in patients with RRMS. These findings suggested an age-related reduction in immune cell penetration into the CSF of PPMS patients (59).

The natural aging of the immune system, known as “immunosenescence”, could be responsible for the shift in MS from an inflammatory to a neurodegenerative nature (60). As we age, the innate and adaptive immune systems undergo numerous changes, including decreased pool of naïve T cells, decreased diversity in T-cell and B-cell receptors, age-related changes in B cell development and function, and accumulation of memory T cells (61, 62). This declining immune functionality, together with other biological processes associated with aging – telomer shortening, DNA mutations, mitochondrial dysfunction, stem cell exhaustion, cellular senescence, or compromised repair capacity of the CNS, among others (63–65) – may contribute to generate the state of chronic low-grade inflammation known as “inflamm-aging”. Inflammaging, coupled with failure of compensatory mechanisms, such as neuroplasticity and remyelination (66), may significantly influence the course of MS and contribute to facilitate a steady neurodegenerative phase in older adults (62). Both inflammaging and immunosenescence can increase susceptibility to infections in older adults (62).

### 1.4 Multimorbidity and polypharmacy

In the aged population, the presence of comorbidities increases, which impacts the disease course, potentially complicating both symptomatology and treatment approaches in MS. Comorbidities, such as infections, cardiovascular and cerebrovascular diseases, diabetes, and psychiatric disorders have been found to be more prevalent in people with MS than the general population (67–69). Older age at diagnosis has been identified as the only factor significantly associated with comorbidities, whereas other factors such as higher baseline disability, higher diagnostic delay, higher relapse rate in the previous year, and lower education were not associated (70). The occurrence of these comorbidities can exacerbate the prognosis of MS and heighten the risk of mortality (71, 72). Comorbidities play a crucial role in deciphering new symptoms in older MS patients, as clinicians must determine whether a decrease in functionality is due to the progression of MS or to a comorbidity. Furthermore, modifiable risk factors such as obesity and smoking have been shown to influence susceptibility to MS (73–78) and disability worsening (73, 79–84).

As a result of multimorbidity, polypharmacy is common in MS (85). Antidepressants, antihypertensives, sedative hypnotics, antiepileptics, antiplatelets, and drugs for peptic ulcer or gastro-esophageal reflux disease are among the most common comedications in these patients (86–88). The odds of polypharmacy were three times higher in those ≥65 years-old than in patients below 50 (86). Among the negative outcomes of polypharmacy are increased fatigue, subjective cognitive impairment, and diminished HRQoL (85). The occurrence of drug-drug interactions has been associated with older age, together with lower educational level (89). These polypharmacy in older individuals is associated with poorer physical and cognitive functioning, even after adjusting for disease burden (90).

Importantly, as patients age, the benefits and risks associated with medications change considerably. Drug metabolism, excretion, and receptor sensitivity may shift as individuals grow older, potentially altering the therapeutic efficacy and side effect profiles of medications (91). Multimorbidity and polypharmacy increase the complexity of MS management and poses major challenges for clinicians and patients (92), despite still remaining underexplored domain in research.

In addition to the challenges posed by comorbidity and polypharmacy, the impact of menopause on women with MS deserves attention. Menopause, characterized by significant hormonal and immunological changes, modifies disease progression and clinical outcomes (93). Studies have observed reduced relapse rates after menopause, whereas the data are inconsistent regarding disability progression, with some studies reporting disability worsening (94) and others observing a similar rate of disability progression (95). Hormonal fluctuations and immunosenescence contribute to neuroinflammation and neurodegeneration, exacerbating MS-related disability post-menopause (96). The concurrent presentation of menopausal symptoms, such as fatigue, cognitive impairments, mood disturbances, and bladder dysfunction, often overlaps with MS symptoms, posing diagnostic and therapeutic challenges (93, 97).

## 2 Managing MS in older individuals

### 2.1 Disease modifying-treatments

#### 2.1.1 Results from clinical trials and real-world evidence

##### 2.1.1.1 Efficacy and effectiveness

The inclusion of older patients in RCT evaluating DMTs for MS has historically been limited, which is a recognized challenge in generalizing trial results to the older MS population. Pivotal trials of the DMTs approved by the European Medicine Agency (EMA) for relapsing MS (RMS) included only patients younger than 55 years (98–108), 50 years (109–112) or even 45 years (113) of age, while studies in SPMS usually include patients up to 60 years of age (114) (**Table 1**). Nevertheless, several subgroup analyses from phase 3 clinical trials have been conducted to understand the influence of age on the DMT efficacy, mainly comparing those aged 18-40 years to the 41-55 years group (104, 106–114, 123, 124). In these post-hoc analysis, significant reduction of clinical and MRI activity in patients >40 years were found with most of the DMTs, including peginterferon beta-1a (125), cladribine (124), dimethyl fumarate (DMF) (104), ocrelizumab (105), ofatumumab (126), natalizumab (123), alemtuzumab (127) and teriflunomide (≥38 years cut-off) (128), although in some studies the efficacy on disease activity in older group was lower than in the younger group (126, 129). With fingolimod treatment the efficacy on relapse rate in patients aged >40 years was present with the dose of 1.25 mg but not with the dose approved of 0.5 mg (100, 107). As far as disability progression is concerned, a statistically significant (108, 124) or numerical (129) lowering of the risk in patients >40 years was observed with cladribine, ofatumumab, and ocrelizumab (108, 124,129) but not with DMF, teriflunomide, natalizumab, and alemtuzumab (101, 104, 123, 128). The significant results cited here were obtained when comparing the DMT with placebo, except for ofatumumab which were obtained when compared with teriflunomide.

**Table 1:** Pivotal clinical trials of disease-modifying Tables therapies for MS.

Name: design (ref)	Evaluated treatment	Upper limit age
Phase III double-blind RCT (113)	Copolymer 1 vs placebo	45 years
PRISMS: phase III double-blind RCT (115)	INFβ-1a vs placebo	50 years
Phase III double-blind RCT (98)	INFβ-1a vs placebo	55 years
Double-blind RCT (109)	INFβ-1b vs placebo	50 years
MIMS: phase III double-blind RCT (99)	Mitoxantrone vs placebo	55 years
Phase III double-blind RCT (116)	Natalizumab vs placebo	50 years
FREEDOMS: phase III double-blind RCT (100)	Fingolimod vs placebo	55 years
TRANSFORMS: phase III double-blind RCT (102)	Fingolimod vs INFβ 1a	55 years
TEMSO: phase III double-blind RCT (117)	Teriflunomide vs placebo	55 years
TOPIC: phase III double-blind RCT (103)	Teriflunomide vs placebo	55 years
TOWER: phase III double-blind RCT (118)	Teriflunomide vs placebo	55 years
CONFIRM: phase III double-blind RCT (101)	BG-12 vs glatiramer acetate	55 years
DEFINE: phase III double-blind RCT (104)	BG-12 vs placebo	55 years
CAMMS223: phase II double-blind RCT (119)	Alemtuzumab vs INFβ-1a	50 years
CARE- MS I: phase III double-blind RCT (112)	Alemtuzumab vs INFβ-1a	50 years
CLARITY: phase III double-blind RCT (120)	Cladribine vs placebo	65 years
OPERA I and OPERA II: phase III double-blind RCTs (106)	Ocrelizumab vs INFβ-1a	55 years
ORATORIO: phase III double-blind RCT (105)	Ocrelizumab vs placebo	55 years
CONSONANCE: phase IIIb open-label (121)	Ocrelizumab	65 years
ASCLEPIOS I and II: phase III double-blind RCTs (108)	Ofatumumab vs teriflunomide	55 years
SUNBEAM: phase III double-blind RCT (107)	Ozanimod vs INFβ-1a	55 years
EXPAND: phase III double-blind RCT (114)	Siponimod vs placebo	60 years
OPTIMUM: phase III double-blind RCT (122)	Ponesimod vs teriflunomide	55 years

ref, reference; interferon beta, INFβ; RCT, randomized controlled trial.

However, it is important to note that these subgroup analyses, in addition to involving patients under 55 years of age, were not powered to detect statistical significance of the differences between the age groups, and therefore these results should be taken with caution. Using a meta-analysis method allows to increase the power to detect differences of DMT efficacy by age. Signori et al. (130) conducted a meta-analysis of six of the above-mentioned RCT with subgroup analysis based on age and found higher reductions of disease activity and disability progression in patients ≤40 years (**Table 2**). This study also reported a significantly higher benefit from DMT on disease activity, but not on disability progression, in patients with baseline gadolinium-enhanced (Gd+) lesions and lower baseline EDSS.

**Table 2:** Meta-analysis of clinical trials assessing the effect of age in DMT efficacy.

Authors (ref)	Aim	Included trials and patients	Main results
Signori et al. (130)	To identify patient subgroups with larger DMT effects.	6 RCT. 6,693 RRMS patients.	DMT efficacy was higher in younger than in older subjects* measured by reduction in ARR (p <0.001) and disability progression (p =0.017).
Weideman et al. (131)	To test whether DMT efficacy in inhibiting disability progression is independent of age.	38 RCT. More than 28,000 MS patients.	The efficacy DMTs on disability strongly decreased with advancing age (p =10^−8^). The regression predicted lack of efficacy beyond ≈53 years. Inclusion of baseline EDSS did not significantly improve the model. HeDMTs outperformed low-efficacy DMTs for patients <40.5 years.
Zhang et al. (132)	To investigate whether age impacts the efficacy of DMTs.	26 RCT. More than 28,000 RRMS patients.	The efficacy of DMTs on reducing ARR, new T2 lesions, and Gd+ lesions was not associated with age.

annualized relapse rate, ARR, EDSS, Expanded Disability Status Scale; DMT, disease-modifying treatment; HeDMT, high-efficacy disease-modifying treatment; leDMT, low-efficacy DMT; MS, multiple sclerosis; ref, reference; RRMS, relapsing-remitting multiple sclerosis; RCT, randomized controlled trial; *patients were grouped in two age groups (‘younger’ versus ‘older’) pooling the estimates collected in the groups as defined in each trial (cut-off point of 40 or 38 years)

Two subsequent metanalysis specifically evaluated the role of age in the efficacy of DMTs for MS in clinical trials (131, 132) (**Table 2**). The first of these meta-analysis, conducted by Weideman et al. (131), assessed whether the efficacy of immunomodulatory DMTs on MS disability progression depended on age. The results showed that DMT efficacy in preventing disability progression strongly decreased with advancing age. Indeed, the model predicted that DMTs had no efficacy on disability after the age of 53 years. Including the EDSS in the model did not change the association between age and DMT efficacy. They also assessed the impact of age in low- and heDMTs, separately, and found that heDMTs were superior to low-efficacy DMTs only in patients younger than 40.5 years. The second meta-analysis adopted the same analytic methods as Weideman et al. but assessed the role of age in the efficacy of DMTs in terms of disease activity (reduction of annualized relapse rate [ARR], new T2 lesions, and Gd+ lesions) instead of disability (132). Here, the authors found no significant association between mean age and the reduction of disease activity, neither clinical nor radiological measures. The authors concluded that despite DMTs being efficient in reducing disease activity regardless of age, patients included in clinical trials were selected based on the presence of baseline disease activity and, therefore, do not represent the real-world patients who have declines in disease activity as they age.

Limitations of these meta-analyses include the absence of access to individual patient data from the trials, with limited information about the age of the participants. Furthermore, there is a notable data gap for patients in their sixth decade and beyond. There is a pressing need for a new meta-analysis that overcomes these shortcomings, aims at assessing both the efficacy of DMTs on disease activity and progression of disability by age. But for that aim, more RCT assessing DMTs in patients older than 55 years need to be conducted.

Indeed, few trials assessing DMTs have included patients older than 55 (114, 133) (**Table 3**); and these trials did not assess the effect of the DMTs by age. The EXPAND trial demonstrated, in patients with SPMS aged 18-60, that siponimod reduced the risk of disability progression significantly more than placebo (114). In the EVOLVE-MS-1, patients with RRMS aged 18-65 had improvements in ARR when treated with diroximel fumarate compared to prior treatments, with no unexpected safety concerns (133). The ongoing trials, CONSONANCE (134) and LIBERTO (135), are evaluating the effectiveness and safety of ocrelizumab in patients aged 18-65 (with PPMS and SPMS in the CONSONANCE trial and with MS in the LIBERTO). Interim analysis of the CONSONANCE study have showed that, over a 2 year period, half of the patients treated with ocrelizumab had no evidence of progression and no disease activity; around one-third of patients had confirmed improvement in at least one disability measure (EDSS, T25FWT, or 9HPT), and most patients (72%) had stable or improved SDMT scores (136). The estimated study completion date is March 2025 for LIBERTO and December 2026 for CONSONANCE. While it remains uncertain if subgroup analyses in patients over 55 from these trials will be conducted, we strongly advocate for them.

**Table 3:** Clinical trials of DMTs including patients with MS over 55 years of age.

Study (ref) - status	Design and treatment	Patients	Primary * and main secondary variables	Age-related findings
EXPAND (114) – completed with results	Double-blind, randomized, phase 3 clinical trial. Siponimod or placebo for up to 3 years or until a prespecified number of CDP.	18–60 years of age, with SPMS and EDSS score of 3.0–6.5.	Percentage of participants with 3-month CDP measured by EDSS. Confirmed worsening of 25FWT; ARR; Gd+T1 lesion, new/enlarged T2 lesion, and brain atrophy.	Siponimod reduced 3-month CDP compared with placebo across age groups, although the treatment effect decreased with increasing age^¥^.
EVOLVE-MS-1 (133) – completed with results	Open-label, phase 3 clinical trial. DRF over 96 weeks.	18–65 years of age, with RRMS and EDSS score of 0.0–6.0.	DRF safety and tolerability. Gd+, new/enlarged T2, new T1 hypointense lesion counts; AR; EDSS; NEDA-3; 25TFW, PROs.	NA
CONSONANCE (134) – ongoing	Open-label, single-arm, phase 3 clinical trial (ongoing). Ocrelizumab over four years.	18–65 years of age, with PMS and EDSS score of 0.0–6.5.	Proportion of participants with NEP. Confirmed worsening of 25FWT; T2 lesion volume; ARR; MSWS-12.	NA
LIBERTO (135) – ongoing	Open label, single arm, extension Phase IIIb/IV clinical trial (ongoing). Ocrelizumab for two years.	18–65 years of age, with MS.	Time to onset of CDP sustained for at least 24 and 48 weeks. Percentage of participants who have CDI, CDP for at least 24 and 48 weeks and over treatment. Percentage of participants who have improved, stable or worsened disability. Mean change from inclusion in parent study in EDSS score over treatment.	NA

ARR, annualized relapse rate; CDI, confirmed disability improvement; CDP, confirmed disability progression; DRF, Diroximel fumarate; EDSS, Expanded Disability Status Scale; Gd+, gadolinium-enhanced; MSWS-12, twelve item MS walking scale; NA, not available; NEDA-3, no evidence of disease activity-3; NEP, no evidence of progression (defined as no progression sustained for at least 24 weeks on all of the following three components: CDP; ≥20% increase in T25FWT; ≥20% increase in 9HPT); PMS, progressive multiple sclerosis; PROs, patient-reported outcomes; ref, reference; RRMS, relapsing-remitting multiple sclerosis; SPMS, secondary progressive multiple sclerosis; T25FWT, timed 25 Foot Walk Test; 9HPT, nine-hole peg test. *The primary endpoint is presented first on the list of endpoints; ^¥^The study was not powered for subgroup analysis.

In general, the selection criteria of RWE studies, are more inclusive, encompassing patients across a wider age range (see **Figure 1**). Studies to evaluate the effect of age on DMT efficacy have been conducted with RWE data. Vollmer et al. re-analyzed data from a retrospective study of MS patients to assess disease activity for oral (DMF and fingolimod) and higher efficacy DMT administered via infusion (natalizumab and rituximab) by age (137). They found that there was a statistically significant difference between oral and infusible DMTs up until the age of 54.2, when disease activity was evaluated according to DMT type (linear model). When disease activity was examined by age subgroup (<45 or >45 years old; binomial model), there was no significant difference in clinical relapses between oral and infusible DMT among those >45 years of age, but patients <45 treated with infusible DMT had significantly lower disease activity. In another RWE study that used linked administrative health data from Canada of over 19,000 patients, treatment with DMT compared with no DMT was associated with a 23% lower hazard of hospitalization in patients <55 year old but not in those over ≥55-years (138). Another Italian retrospective study included RRMS patients with ≥5 years follow-up and ≥3 EDSS stratified by age (≤18 years; 18–49; ≥50). The authors found that sustained exposure to DMT decreased the risk of disability progression; the effectiveness of DMT was lower in LOMS, although still detectable (139). Similar effectiveness has been observed for injectables and oral first-line DMTs in patients with LOMS (RRMS) in terms of first relapse, time to confirmed disability progression (CDP), and time to discontinuation (140).

RWE on the effectiveness of specific DMTs in older patients has also been published (141–145). One study conducted during COVID-19 pandemic aimed at understanding ofatumumab utilization patterns and patient characteristics reported that one-third of patients treated in the real-world were ≥55 years (141). However, the study neither provided the results by age, nor reported effectiveness or safety outcomes.

The effectiveness of interferon beta (IFNβ), a DMT commonly used in older MS patients, have also been assessed in the real world. One prospective 2-year study showed that patients older than 50 years treated with INFβ had decreases in the ARR and stable anxiety and depression (142). Other retrospective study reported no significant association between exposure to IFNβ and disability progression in this population (39).

A recent retrospective study, using data from the Italian MS Registry, examined the effectiveness of ocrelizumab in PPMS patients not meeting or meeting the ORATORIO eligibility criteria (aged 18-55 years, EDSS of 3.0-6.5, a disease duration <15 years if EDSS >5.0 or <10 years if EDSS ≤5.0) (143). Patients in the non-ORATORIO group were further stratified according to age (≤55, 56–64 and ≥65 years), EDSS (≤ 6.5 and >6.5), and disease duration (≤10–15 and >10–15 years). In patients not meeting the ORATORIO criteria, those aged over 65 years at ocrelizumab initiation demonstrated a significantly higher confirmed EDSS worsening of ≥1 point at 12 months and of ≥2 points at both 12 and 24 months, compared to patients between 56-64 years and ≤55 years old. Another retrospective study that included patients older than 55 with PPMS or SPMS at ocrelizumab initiation found that 60% of patients remained stable or improved after 2 years of ocrelizumab treatment (144).

The effectiveness of cladribine, measured as time to evidence of disease activity, in patients aged 50 years and older has been observed to be comparable to that in younger patients (under 50) over a median follow-up period of 12.4 months (145).

##### 2.1.1.2 Safety

Alterations in the immune system due to biological aging heighten susceptibility to infections, neoplasms, and lymphopenia (146–148). These alterations, combined with the changes induced by DMTs on the immune system, have been hypothesized to produce synergistic effects (149). Older individuals are generally more susceptible to severe adverse events (AEs), such as the risk of progressive multifocal leukoencephalopathy (PML) (150). The increased risk in PML is because the prevalence of seropositivity to the John-Cunningham virus (JCV) – which causes PML – grows with age. This condition has been primarily linked to natalizumab (151, 152), but also to the use of other DMTs such as fingolimod (153), especially after previous treatment with natalizumab (154), and DMF (150). Older age has been observed to independently heighten the PML risk, with these patients being more prone to develop PML after fewer natalizumab infusions and exhibiting higher mortality rates (155).

Other infectious complications seem to also be related to aging (156). Older patients were more susceptible to cryptococcal meningitis associated to fingolimod (157), infections requiring hospitalization associated to ocrelizumab (158), and herpes zoster across several DMTs (149). Among MS inpatients with COVID-19, those receiving DMT —except for those treated with anti-CD20 monoclonal antibodies— had reduced odds of 30-day mortality and a lower risk of death (159). Nevertheless, older age, progressive MS, comorbidities and higher disability were associated with worse COVID-19 outcomes in patients on DMTs (159–161). Sustained treatment with anti-CD20 might not be a feasible choice in older MS patients due to a reported heightened risk of severe and opportunistic infections, malignancies, and a diminished vaccine response associated with these treatments (162).

A meta-analysis with meta-regression of 45 RCTs was conducted to investigate if there was an age-related rise in infections and neoplasms among MS patients receiving DMTs with different mechanisms of action regarding the effects on peripheral leukocytes: 1) immunomodulatory (DMF, glatiramer acetate, INFβ, and teriflunomide), 2) sequestrating (fingolimod, natalizumab, ozanimod, and siponimod), and 3) depletive (alemtuzumab, cladribine, and ocrelizumab) (163). The mean age in the clinical trials was 38 years. The investigators found that age alone did not influence the rate of any AEs, but interaction of age with depletive mechanism of action explained approximately 23% of the variance in neoplasm rate. In patients older than 45 years treated with depletive agents, the risk of neoplasms was found to be higher compared to other treatments. Conversely, there was no association between age and the rate of overall infections and herpetic infections.

A prospective real-world study conducted in Germany included patients treated with teriflunomide and analyzed the data based on age subgroups. Results on safety showed that the number of patients with AEs was lowest in patients aged 26–35 years but was not different in the rest of age groups (18–25, 36–45, 46–55, 56–65 and 66+ years). The rate of patients with infections was similar regardless age (≤45 and ≥45 years). However, the rate of discontinuations was significantly higher in patients aged >45 years (62.9%) than in the younger group (37.1%). Further research is needed to determine the safety of specific DMTs in older patients with MS, including those with LOMS (164).

In a real-world study in the United States, patients aged18 to 74 years who switched from high-efficacy infusion DMTs to oral cladribine did not experience any new AEs (165). When comparing older (≥50 years) and younger (<50) patients treated with cladribine, AEs and infections during treatment were numerically more frequent in older patients (145), especially among those who experienced Grade ≥3 lymphopenias (166); however, these differences were not statistically significant (145, 166).

#### 2.1.2 Initiation, de-escalation and discontinuation

Initiating DMTs in individuals with MS older than 55 years has been a subject of debate among clinicians. The decision to start, continue, or switch DMTs in older individuals requires a deep understanding of the benefits and risks in this age group, where scientific evidence is limited, as we have discussed in the prior sections.

Information on prescribing patterns of DMTs among older adults with MS remains scarce. A study conducted in the US included 12,922 older adults with MS showed that around 19% of patients were receiving a DMT (10.5% injectables, 6% orals, and 2.5% injectables). Older adults aged 60-69 years had higher odds of receiving DMT than adults aged ≥70 years. Several factors increased the odds of being prescribed a DMT, such as the presence of symptoms or symptomatic medication (167). Many experts are advocating for the early initiation of heDMT in MS (168–173), based on the increasing evidence supporting the benefits of this strategy in controlling disease activity and progression (15, 174, 175). The strategy seizes the window of opportunity during which the underlying pathophysiology can be altered and influence long-term clinical outcomes (176). Whether heDMT should be use as early as possible in older patients or if moderate efficacy DMT are a better option for this population has not been specifically evaluated.

A Danish study comprised all patients (N=3497) with RRMS registered in The Danish Multiple Sclerosis Registry who began a DMT after 2014. After mutual adjustment for all selected covariates (DMT efficacy, pre-treatment relapse activity, disease duration, EDSS score, and MRI activity), patient age was a strong decisive factor for choosing a heDMT with odds ratio 1.69 for starting a heDMT in patients <40 years compared with patients ≥ 40 years (177).

Some authors have stated that in PPMS patients older than 55 years, with a long disease course (> 15 years) and a high degree of disability (EDSS score > 6.5) therapeutic nihilism (i.e. avoiding DMT use) should be avoided (171).

Two pragmatic clinical trials are currently being conducted to delve deeper into escalation and early heDMT: DELIVER-MS (NCT03535298) and TREAT-MS (NCT03500328). Despite the anticipated valuable insights that these two studies will provide concerning treatment strategies in MS, it is noteworthy that both studies limit their patient cohorts to a maximum age of 60 years. Consequently, while the data derived from these studies will shed light on treatment approaches for patients aged between 55 and 60 years, they leave patients ≥60 uncovered. Further research specifically addressing treatment strategies for MS patient older than 55 years is still needed.

Weideman et al, considering the results of their meta-analysis (after age 53 there was no predicted benefit to receiving DMTs) stated that “a prescribing clinician must consider the possibility that starting or continuing immunomodulatory DMT beyond age 53 will expose an average patient to treatment-associated risks with few, if any, potential benefits”, although they recognized that the model was based on average outcomes, and individual genetic and environmental factors were not considered (131).

The optimal duration of treatment with a DMT still remains under debate, and the available evidence regarding de-escalation or discontinuation is still limited, even if we are now encountering aging patients who have been treated with DMT for decades (178, 179). The DISCOMS was a phase 4 non-inferiority RCT aimed to assess whether it was safe to discontinue DMT in aged MS patients. The study included people with any MS subtype, ≥55 years old, with no relapse within the past 5 years or new MRI lesion in the past 3 years while continuously taking an approved DMT. Participants were randomly assigned (1:1) to either continue or discontinue DMT. Similar numbers of patients had AEs and serious AEs (SAEs), but a higher number of AEs and serious SAEs occurred in the discontinue group. The authors concluded that the null hypothesis could not be rejected, and they could not determine if discontinuing DMTs is equally safe compared to maintaining treatment in patients over 55 years without recent disease activity (180).

Studies from the real-world setting have explored the association between DMT discontinuation and both disability progression and disease activity. A registry-based study, with an average follow-up of 4.6 years, revealed that prior to DMT discontinuation, 72.5% of patients were classified as stable (<1.0 or <0.5 increase if EDSS<6.0 or ≥6.0, respectively). However, after discontinuation, 32.9% of these previously stable MS patients experienced disability progression. Two years after DMT discontinuation, the progression rate was comparable for patients younger (31.1%) and older (25.9%) than 55 years old (181). Another study evaluated disease activity in RRMS patients who discontinued first-line DMT. The study showed that higher age at DMT discontinuation (45-55 vs <45 years) was associated with a lower risk of MRI activity and relapses after discontinuation (182).

Other studies have explored disease activity and disability progression in older adults, but without comparing the results with a group of younger patients. For instance, one study conducted in France in RRMS patients over 50 years with a median follow-up of 7 years after DMT discontinuation showed that discontinuation was not associated with an increased risk of relapse or EDSS progression, but was associated with a higher risk of reaching an EDSS of 6 (183). Another study assessed inflammatory activity after the discontinuation of second and first-line DMT in patients over 45 years. Within a year of discontinuation, they discovered that the likelihood of experiencing a relapse was considerably higher for natalizumab than for first-line DMT or fingolimod (184). Similar findings were obtained in a prior study where discontinuation of natalizumab, but not first-line DMTs, was associated with rebound (185). In patients with SPMS, the ARR remained stable after DMT discontinuation (186). These data suggest that disability progression after DMT discontinuation is not significantly influenced by age, but disease activity might be, depending on the DMT. Thus, when prevention of both relapses and disability worsening is the goal, age is a complex variable to consider.

Fewer studies addressed the de-escalation phase, the transitionary from heDMT to lower efficacy DMT. A recent retrospective study evaluated patients who switched from a heDMT — including any monoclonal antibodies or oral DMTs, except teriflunomide — to a low-efficacy DMT. Using a noninferiority analysis, the study found that de-escalating from a heDMT to a low-efficacy DMT did not worsen ARR or disability, although these findings were not significant; the only significant observation was that the T2 lesion burden did not worsen when switching (187).

There is a lack of evidence-based guidelines for optimal initiation, de-escalation or discontinuation of DMT in MS patients older than 55 years (188, 189). In the absence of evidence-based guidelines and consensus, it is essential to note that the decision to initiate DMTs in older MS patients should be individualized. Some older patients might still have active inflammation and might benefit from treatment. Also, newer DMTs with different mechanisms of action might offer advantages in terms of efficacy and safety profiles for older individuals. Therefore, while age is an essential factor in the decision-making process, the overall clinical picture, including disease activity, progression rate, comorbid conditions, and patient preferences, should guide the choice of initiating, de-escalating, or discontinuing treatment.

Overall, given the safety concerns associated with DMTs in older patients, coupled with the heightened prevalence of comorbidities in this demographic, the primary factor in deciding to use a DMT in older patients should be safety rather than efficacy.

#### 2.1.3 New treatments: BTKs

Despite current DMTs effectively reduce relapses and associated disability worsening, mainly linked to immune cells infiltrating the CNS, they are less effective in slowing overall disability accumulation, possibly due to not targeting inflammation inside the CNS, a key driver of disability. BTK (Bruton tyrosine kinase) is an intracellular signaling molecule involved in the regulation of maturation, survival, migration and activation of B cells and microglia (190). Although BTK inhibitors were originally developed for B-cell malignancies, such as certain types of lymphomas and leukemias, they have been explored as potential treatments for MS due to their ability to modulate B-cell activity and other immune responses. The second-generation BTK inhibitors being explored as potential treatments for MS have improved their safety profile resulting from reduced off-target inhibition of molecules such as Janus kinase 3, epidermal growth factor receptor, and possibly other TEC tyrosine kinases family members (191).

Phase II safety and efficacy data for evobrutinib and tolebrutinib have been published (192, 193), but only the evobrutinib trial included patients up to 65 years old. In the overall population, the common AEs were nasopharyngitis and elevated liver enzymes (192). By week 52, higher doses of evobrutinib had greater AEs rates. An open-label extension following the initial study indicated that most participants continued with the treatment, and the initial data did not raise new safety concerns. No results by age subgroup are available so far. Unfortunately, in the phase III trial comparing evobrutinib and teriflunomide, evobrutinib did not meet their primary endpoints of reducing ARR in people with RMS compared to oral teriflunomide (194). Only some of the ongoing phase II trials of tolebrutinib and fenebrutinib include older patients (up to 60 years [NCT04411641], 65 years [NCT04544449] or 120 years [NCT04742400]).

#### 2.1.4 Treatments focused on remyelination and neuroprotection

The loss of myelin sheath, that characterizes MS, disrupts not only the efficient transmission of nerve impulses but also the preservation of the integrity of the axon (195). Following demyelination, the unprotected axons are vulnerable to energy deficits, potentially leading to neurodegeneration that might be irreversible. Consequently, the regeneration of new myelin sheaths through remyelination after a demyelinating event is crucial to maintain axonal stability and prevent the major pathological change underlying the progressive phase of the disease.

Oligodendrocytes (OL) newly generated from oligodendrocyte precursor cells (OPCs) were proposed to be responsible for remyelination in the adult human CNS (196, 197). Upon receiving signals from a lesion, these OPCs differentiate into OL, which then begin to wrap around the denuded axons, forming new myelin sheaths. Also, recent studies showed that mature OL can regenerate new myelin sheaths after demyelination (198). In individuals with MS, remyelination is often incomplete or insufficient, particularly at older age (199), leading to persistent functional deficits and leaving axons vulnerable to degeneration. The incomplete remyelination in MS can be attributed to various factors (200). With age and disease progression, the reservoir of OPCs can diminish, and their differentiation into myelin-producing OL can be hindered (201, 202). Furthermore, the MS lesion environment, replete with inflammatory molecules and scar tissue, may be hostile to the remyelination process (200).

Strategies for remyelination are diverse and encompass the enhancement of oligodendrocyte progenitor cell mobilization (via semaphorins) and differentiation (through pathways such as Notch, LINGO-1, and Wnt; or agents like vitamin D, thyroid hormone, or activation of the retinoid X receptor). Other tactics involve the stimulation of neuronal activity or the modulation of microglial targets (203). **Table 4** presents late-stage clinical trials evaluating agents targeting remyelination that included MS individuals older than 55 years.

**Table 4:** Phase III and IV clinical trials of remyelination treatments including patients older than 55 years.

Agent (ref)	Design/patients	Primary outcome result/status
Adrenocorticotropic Hormone (NCT02446886)	Phase IV, randomized, open-label trial. RRMS or SPMS; ≥18 years old.	Completed. No publications available
MD1003 (high-dose biotin) (204)	Randomized, double-blind, parallel-group, placebo-controlled trial. PPMS or SPMS; 18-65 years old.	39 (12%) of MD1003-treated patients achieved disability reversal at month 12, confirmed at month 15, vs 29 (9%) of the placebo-treated patients (not significant).
MD1003 (high-dose biotin) (MS-ON) (205)	6-month, randomized, double-blind, placebo-controlled study. MS; 18-75 years old.	The mean change in VA was not larger with MD1003 than with placebo (p = 0.66).

ref, reference; RRMS, relapsing-remitting multiple sclerosis; RSPMS, relapsing secondary progressive multiple sclerosis; SPMS, secondary progressive multiple sclerosis; VA, visual acuity.

Some agents such as biotin showed promising results in terms of disability reversal in small trials (206), but failed to replicate its efficacy in trials with a higher number of patients and more ambitious endpoints (204). Phase I and II trials have been performed in MS patients older than 55 years evaluating agents such as liothyronine (207), olesoxime (208), clemastine fumarate (209), quetiapine (210), opicinumab (211, 212), rHIgM22 (213), domperidone (214), amiloride, fluoxetine, riluzole (215), and elezanumab (216). Several of these studies did not meet the primary endpoint (208, 210–212, 215) and further phase III trials were not encouraged. In those trials where the study endpoints were met (207, 209, 213), phase 3 trials will need to confirm the efficacy and safety of these agents. The MACSiMiSE-BRAIN is an ongoing phase II trial conducted to assess the neuroprotective and remyelinating effects of metformin in MS patients of 18-70 years. The study is estimated to be completed in 2026 (217). Up to date, new remyelinating and neuroprotective agents have not yet successfully showed results in older MS patients.

### 2.2 Symptomatic treatments

Symptomatic treatments focus on alleviating symptoms that patients experience throughout the course of their illness. Given the heterogeneity of MS symptomatology, a tailored, patient-centered approach is essential in symptomatic management. Available symptomatic treatment encompasses pharmacological and non-pharmacological interventions, such as physical and occupational therapy, psychotherapy, and lifestyle modifications. Here we present a review of the evidence of the effectiveness of these treatments in studies that included older patients.

#### 2.2.1 Pharmacologic treatment

The pharmacologic treatment approach for each symptom can vary based on the prevalence of symptoms and range of available therapeutic options. Typically, pharmacological therapy are combined with non-pharmacological strategies, as a combined approach often yields better outcomes (218). A comprehensive study from Germany, encompassing both pharmacological and non-pharmacological treatments, assessed 35,755 MS patients. This study showed that most of the patients (up to 80%) with depression, spasticity, pain, and epilepsy were treated. Contrastingly, only a minority (21-36%) received treatment for fatigue and cognitive dysfunctions, a gap attributed by the authors to the scarcity of treatment options (17). Additionally, a Canadian survey reported even higher treatment rates for depression, at 85.7%, covering also both pharmacological and non-pharmacological interventions (219).

Several pharmacological agents are used to treat the symptoms of MS, but for most of these treatments, the evidence supporting their efficacy in patients with MS is weak. Several meta-analyses have been conducted, examining both RCT and uncontrolled studies, to assess the effect of these treatments in MS symptoms. To date, while no meta-analysis has explicitly focused on the impact of these treatments in older patients, each one has incorporated studies with patients aged 55 or over. **Table 5** presents pharmacologic treatments that, based in meta-analysis findings, have shown consistent efficacy in alleviating various symptoms. These include fampridine, cannabinoids, botulinum toxin, nabiximols, desmopressin, and amantadine (220–233).

**Table 5:** Meta-analysis showing efficacy of symptomatic treatment in MS.

Symptoms	Pharmacological treatment
Impaired gait	Fampridine (220)^*^ (221),^*^ (222),^*^ (223),
Spasticity	Cannabinoids (224)^*^ (225),^*^ (226), ^*^
	Botulinum toxin (224)^*^
	Nabiximols (227)^*^
Pain	Cannabinoids (225)^*^
Bladder dysfunction	Cannabinoids (225)^*^
	Desmopressin (228)^*^
Fatigue	Amantadine (229) (230),^*^
Cognitive dysfunction	Fampridine (220)^*^ (231),^¥^
Finger dexterity	Fampridine (220)^*^
Tremor/ataxia	Botulinum toxin (232)^*^ (233),^*^

*Significantly higher efficacy compared to placebo in RCT; ^¥^Only when assessed with the symbol digit modalities test (SDMT).

Other symptomatic treatment approaches have reported positive results in MS patients, although they have not been confirmed by meta-analysis. For spasticity, other treatment options include baclofen, tizanidine, and gabapentin as first line (234–236) and dantrolene or diazepam as second-line (236). Consensus documents on therapeutic management of spasticity in patients with MS are available (237).

Pain management has involved the use of tricyclic antidepressants, serotonin noradrenaline reuptake inhibitor (SNRI), carbamazepine, gabapentin, pregabalin, and lamotrigine (235, 238, 239). Duloxetine has been recognized for its potential to treat both depression and fatigue, while fluoxetine and bupropion have been noted for their effects on depression, with the latter also addressing sexual dysfunction (238, 240–242). In treating tremor, agents such as propranolol, primidone, anticholinergics, gabapentin, topimarato and clonazepam are used (243). Bladder dysfunctions has also been addressed using mirabegron, antimuscarinic agents (solifenacin, tolterodine, trospium) and botulinum toxin (244–249).

Also, it is important to note that not all studies of pharmacological symptomatic treatments have demonstrated efficacy in patients with MS. Some studies have reported a lack of positive effects or inconsistent results of some pharmacologic treatments for ataxia or tremor (233, 250). Also, the efficacy of oxybutynin for bladder dysfunction (251) and amantadine, pemoline, modafinil, 4-aminopyridine, and prokarin for fatigue (252, 253) remains contentious. Desipramine and paroxetine showed a trend towards efficacy in treating depression in MS, but the trials assessing these antidepressants had a high number of patients lost to follow-up (254). Also, vortioxetine was effective in reducing the symptoms of depression and anxiety, but it did not improved cognition or fatigue in a recent case series (255). Results from a meta-analysis showed that acetylcholinesterase inhibitors (AChEIs; donepezil and rivastigmine) and stimulants (methylphenidate, modafinil, l-amphetamine sulfate and lisdexamfetamine dimesylate) offered no significant benefits over placebo in cognition – specifically in processing speed, verbal fluency, working memory, verbal and visuospatial memory or executive function – (256). Also, there is evidence that drugs with sedative or psychotropic properties, such as benzodiazepines, cannabinoids and anticholinergics, can potentially compromise cognitive or physical functioning (257–259).

Few studies have investigated the effect of age on the safety of symptomatic treatments in MS. In a large study, it was observed that MS patients aged 50-64 had twice the likelihood, and those 65 and older had over three times the likelihood, of being on multiple medications compared to younger patients (86), which is known to increase the risk of AEs. *Post hoc* analyses have revealed that older age does not affect response or AEs associated with duloxetine; but combining duloxetine with pregabalin or gabapentin with imipramine, nortriptyline or venlafaxine might offer added benefits over monotherapy, they also pose a heightened risk of AEs (260). Despite studies having included adults of a wide age range, research assessing the effect of these treatments by age is still missing. There is a need for studies that evaluate the safety and efficacy of medications targeting symptoms, particularly when used in combination, in older MS patients.

##### 2.2.1.1 Treating symptoms clusters

Most MS treatments focus on individual symptoms, often leading to polytherapy, which heightens the risk of AEs, drug interactions, and can intensify other MS symptoms. Simplifying MS symptom management would be beneficial for patients. An approach to do this is by pinpointing symptoms with a shared pathological mechanism or those treatable with a single therapy. This reasoning led to the Spasticity-Plus syndrome concept, which groups MS spasticity-related symptoms with similar pathophysiology or treatment response (48). Two major clusters have been identified: spasticity-spasms/cramps-pain, and ataxia-instability-vertigo. Depression, cognitive impairment, and fatigue were clustered by common pathophysiology. A common underlying pathophysiology between spasticity and associated symptoms could explain the fact that pharmacological intervention targeting the cannabinoid system, such as nabiximols, has effects on several spasticity-associated symptoms (48).

##### 2.2.1.2 Vaccination

Given that some DMTs may lead to increased rates of infection-related adverse events and considering that older adults inherently have a higher risk of infections, routine assessment of vaccination status in older patients with MS is very important to allow updating of vaccines as needed. Vaccination against common and potentially severe infections such as hepatitis, diphtheria, tetanus (often combined in the DT vaccine), influenza, COVID-19, and pneumococcal disease is recommended (261).

#### 2.2.2 Non-pharmacologic approaches

In the older population, where comorbidities and polypharmacy are more prevalent, non-pharmacologic interventions offer unique benefits (262–265), often complementing drug treatments. Symptoms such as spasticity, impaired gait, cognitive decline, fatigue, and sexual dysfunction can be intensified by age-related changes in the musculoskeletal, nervous, and sensory systems, leading to a more pronounced decline in overall function and mobility.

##### 2.2.2.1 Physical therapy and exercise

Both physical therapy (PT) and physical exercise play crucial roles in the management of MS patients (48, 265, 266). PT offers tailored interventions that are delivered by trained professionals, and usually targets a specific symptom. Several meta-analyses (**Table 6**) have concluded that PT interventions are effective for improving gait and balance and addressing spasticity, sexual dysfunction, and bladder issues (268–272, 276, 277). Also, the benefits of PT for improving gait, balance, fatigue and QoL appear to be enhanced when incorporating robot-assisted training or virtual reality tools (267, 268, 275, 278, 289, 290). Moreover, physiotherapy may enhance the benefits of symptomatic drugs, such as botulinum toxin for spasticity (291).

**Table 6:** Meta-analysis of physical therapy and physical exercise in MS symptomatology.

Intervention	Studies and patients	Main findings/Conclusions
PT for gait impairment (267)	11 studies (number of RCT and patients not reported). ≥18 years old, with severe mobility disability (EDSS score ≥6.0 or mobility disability). Description of age or EDSS NR.	RAGT improves performance on the 6MWT, 10MWT, fatigue severity scale, and BBS. Body weight-supported training and traditional walking training fails to yield significant improvements in mobility-related outcomes.
PT for spasticity (268)	25 studies (16 RCT), 799 patients with MS. Mean EDSS range: 2-7.5. Description of age NR.	The most compelling evidence is for the positive impacts of exercise therapy. RAGT alleviates self-reported spasticity and outpatient exercise programs has a positive impact on muscle tone.
Aquatic PT (269)	6 RCT, sample size varied from 23 to 73 patients with MS. Mean age range: 30.4-52 years. Description of EDSS NR.	Aquatic PT improves balance, fatigue, and functional capacity.
PT for sexual dysfunction (270)	8 studies (7 RCT; number of patients NR). Description of age or EDSS NR.	PT interventions provide clinically and statistically significant improvements in sexual function, satisfaction, and emotional well-being. Notably, PFMT and mindfulness are among the most effective strategies in improving sexual function and sexual satisfaction.
PT for risk of falls (271)	16 RCT, 724 patients with MS. Mean age range: 35.3-60.0. Mean EDSS range: 0-7.	The evidence, ranging from very low to moderate quality, suggests that the effectiveness of PT interventions in reducing falls is limited. However, home-based exercises appear to have potential in decreasing falls for ambulatory patients.
Vestibular training for balance impairments and dizziness (272)	5 RCT, 321 patients with MS^¥^. EDSS ≤7. Mean age: 43.6 years^¥^.	Vestibular rehabilitation proves more effective in enhancing balance and alleviating dizziness symptoms than no intervention. Compared with other exercise interventions, improvements in favor of the experimental group were observed, but differences between groups were not statistically significant.
Exercise, yoga, and PT for QoL (273)	18 studies (13 RCT), 725 patients with MS. Description of age or EDSS NR.	Aerobic exercise and PT effectively improve satisfaction in physical, mental, and social functioning. Yoga and combined exercises show a significant impact on any QoL domains.
Yoga, ICF (274)	15 studies (13 RCT), 827 patients with MS. Description of age or EDSS NR.	Yoga performs similarly to other exercise methods when used as a treatment for MS in the domains of the ICH model.
PT with VR for balance impairments and risk of falls (275)	14 studies, 663 patients with MS^¥^. ≥18 years. Description of age or EDSS NR.	VRBT show better results in enhancing balance and reducing fall risk in individuals with MS compared to no intervention or conventional rehabilitation, though the certainty of evidence is very low for both comparisons.
PFMT for lower urinary tract dysfunction (276)	9 studies (RCT) Mean age range: 36.3-52.1. Mean EDSS range: <7.	Moderate to high-quality studies show the overall efficacy of PFMT in decreasing urine leakage and neurogenic bladder symptoms and increasing endurance and power of pelvic floor muscles.
PTNS and PFMT for neurogenic bladder or neurogenic lower urinary tract dysfunctions (277)	6 studies Description of age and EDSS NR.	PTNS improved daytime frequency, nocturia, urgency episodes, voided volume, and urge incontinence. PFMT improved endurance and fast contraction components of the PERFECT scheme.
PT with VR for balance impairments, fear of falling and gait speed (278)	19 RCT, 858 patients with MS. Mean age of 43.4years. Mean EDSS: 3.6.	VRBT is effective in improving balance and in reducing fear of falling, but not on gait speed.
PT for walking (279)	21 RCT, 947 patients with MS. Mean age of 44.5 years. EDSS <6.5.	PT yielded a small, but statistically significant overall improvement in walking outcomes compared with usual care.
Exergames for cognitive function (280)	13 studies (9 RCT), 465 patients with MS, post-stroke hemiparesis, PD, dementia, dyslexia, or Down syndrome. Mean age range (MS): 35.1-50.0.	Exergames significantly improve executive functions and visuo-spatial perception when compared to the alternative or no intervention. There are no significant differences for attention and global cognition.
Exercise for depressive symptoms (281)	13 RCT, 447 patients with MS. Mean age range: 35.2-55.2 years. Mean EDSS range: 2-8.1.	Exercise training result in a significant improvement in depressive symptoms compared to control.
Exercise for depressive symptoms (282)	12 RCT, 476 patients with MS. Mean age range: 36.4-62.0. Mean EDSS range: 0-8.	There is a small but positive effect of exercise on depressive symptoms. However, the studies were very heterogenous in terms of exercise intervention, instrument to assess depression, baseline symptoms, and disability level.
Physical exercise for fatigue (283)	58 RCT, 2644 patients with MS. Mean age range: 41.5-50.7 (age range: 29-61.6), mean EDSS range: 2.7-6.1.	Among the different exercise modalities, combined exercise is the most effective exercise for alleviating both physical and total fatigue. Resistance training is also effective for total fatigue.
Physical exercise for fatigue (284)	31 studies, 1434 patients with MS. Mean EDSS range: 0-5. Description of age NR.	Physical exercise significantly reduces fatigue in patients with MS.
Physical exercise for QoL, depressive symptoms and cognition (285)	122 studies (MS=35), 7231 patients with MS, AD, HD, PD, Sz or UD. Mean age range: 15.4-84.0. Description of EDSS NR.	Exercise is an efficacious and safe supplementary therapy for several chronic brain disorders. It demonstrates a medium-sized effect on QoL and a large effect on mood, with a positive dose–response correlation. Exercise also improved several cognitive domains with small but significant effects.
Exercise for anxiety (286)	4 RCT, 133 patients with MS. Mean age range: 35.2-51.6. Mean EDSS range: 2.2-6.0.	Exercise appears to have no significant impact on anxiety in individuals with MS. However, these findings should be taken with caution. The studies exhibited potential biases and small sample sizes, and did not include anxiety as the primary endpoint.
Pilates for physical function (287)	6 studies (5 RCT), 247 patients with MS. Mean age range: 26-54.6. Mean EDSS range: 1.75-4.6.	Pilates improves physical function and might be helpful for reducing self-perceived fatigue, and increasing balance confidence and walking ability. The effects of Pilates are not significantly greater than those derived from other physical therapies.
Yoga for fatigue, mobility and HRQoL (288)	9 studies (7 RCT), 670 patients with MS. Mean age range: 31.6-54.4 years. EDSS NR.	In the short term, yoga demonstrates benefits over usual care in alleviating fatigue and mood, though no notable differences were observed for HRQoL, muscle function, or cognitive function. When comparing yoga to traditional exercise, no significant short-term or long-term effects were observed.

AD, Alzheimer’s disease; BBS, Berg Balance Scale; EDSS, Expanded Disability Status Scale; HD, Huntington’s disease; HRQoL, health-related quality of life; ICH, International Classification of Functioning, Disability, and Health; NR, not reported; PD, Parkinson’s disease; PFMT, pelvic floor muscle training; PT, physical therapy; PTNS, peripheral tibial nerve stimulation; QoL, quality of life; RAGT, robot-assisted gait training; RCT, randomized controlled trial; Sz, schizophrenia; UD, unipolar disorder; VR, virtual reality; VRBT, virtual reality-based therapy; 6MWT, 6-minute walk test; 10MQT, 10-metre walk test

^¥^data combined from the RCT and two additional studies not included in the meta-analysis (quantitative synthesis) but included in the systematic review (qualitative synthesis).

On the other hand, exercise training, including endurance and resistance training, can be part of patients’ daily or weekly routine. Exercise training has been also proved by meta-analyses to reduce fatigue and symptoms of depression (281, 282, 284, 292, 293) and to improve balance and HRQoL (294, 295). Although the studies included in the meta-analyses assessing the effect of PT and exercise did not specifically target patients older than 55 years, most of the studies did not impose an age limit in their selection criteria, leading to a broad age range among participants (**Table 6**).

Individuals with MS, including older adults, exhibit reduced physical activity levels compared to the general population, underscoring the imperative for targeted physical activity promotion within these patients (296–299). Interventions to increase physical activity behavior among people with MS are efficacious for increasing and sustaining physical activity behavior (300). Resistance training, both in isolation and combined with aerobic exercise, has proven most effective for enhancing muscular fitness, while endurance exercises improve cardiorespiratory fitness (301).. In older adults with MS, targeting moderate-to-vigorous physical activity as an approach for improving cardiorespiratory fitness has been suggested (302).

Importantly, physical exercise has been shown to increase neuroplasticity (303), even in the aging brain (304). A comprehensive meta-analysis revealed that while low and high-intensity exercise improved neuroplasticity, the relationship between exercise intensity and neuroplasticity seems to be present in younger adults but not in older adults or patients with MS, Parkinson’s disease (PD) or stroke (305). Thus, exercise intensity is a crucial factor to adjust the exercise regimen for healthy young individuals, but it may not be as critical for older adults with MS (305). The meta-analysis also concluded that exercise-induced improvements in neuroplasticity were associated with motor changes but not cognitive changes (305).

It is important to note, however, the inconsistent reporting of age in the meta-analyses conducted to assess the effect of physical therapy and physical exercise in MS symptomatology (see **Table 6**). In some of these meta-analyses, data on age was not reported (267, 268, 270, 273, 274, 277, 284). The lack of standardization in reporting the age of the patients included in the studies poses a challenge to interpreting and comparing findings across studies. Standardizing the reporting of age data would facilitate drawing conclusions from these meta-analyses.

##### 2.2.2.2 Neuromodulation therapies

Neurostimulation, which involves using cutting-edge technologies for stimulation or inhibition of the neural function, has received limited attention in MS, compared to other neurological diseases. Some studies in a wide age range of the MS population have been conducted.

A research study encompassing participants aged between 27-75 demonstrated the long-term efficacy of the intrathecal baclofen pump (ITB) in diminishing symptoms of spasticity, spasms, depression, and pain, while also enhancing overall function. Furthermore, the safety of ITB remained consistent, unaffected by the age (257). Functional electrical stimulation (FES) has shown an initial and persistent benefit on gait speed specifically with short walking evaluations (306). Deep brain stimulation (DBS) has been observed to alleviate MS-related tremor (307, 308). Though DBS has not specifically been studied in older MS patients, results from PD research revealed that DBS improves motor function and reduces medication reliance in PD patients, including those over 70 (309). Transcranial direct current stimulation (tDCS) seems to improve processing speed, mood, pain, and fatigue (310). Also, spinal cord stimulation (SCS) seems beneficial for MS-associated pain and bladder issues but its efficacy for spasticity is mixed (311, 312). Bladder disorders seems to benefit from peripheral tibial nerve stimulation (277). Additionally, repetitive transcranial magnetic stimulation (rTMS) shows preliminary evidence of efficacy in improving spasticity in MS patients (313), though more studies are required to assess its impact on fatigue (314). These insights underscore the potential of diverse neuromodulatory approaches in addressing a range of MS symptoms.

##### 2.2.2.3 Psychotherapy, cognitive rehabilitation and mindfulness

Psychological interventions have demonstrated efficacy in enhancing the mental health, sexual dysfunction and HRQoL of patients with MS (46, 315–318). The efficacy of psychotherapy in MS extends beyond in-person sessions, including sessions conducted via telephone or online platforms (319, 320). Cognitive-behavioral therapy (CBT) is especially prominent due to its targeted approach in helping patients manage their pain and stress and in reducing symptoms of fatigue and depression (315, 321–327), although some researchers have reported CBT not to be efficacious when considered alone (328).

Patients with MS also benefit from cognitive rehabilitation programs (329, 330). Memory, attention, and executive function deficits have showed some improvements with cognitive rehabilitation (330–332). However, the evidence is still limited (330) and most RCT did not properly report information regarding the content or procedures of the intervention (333). There remains a critical need for large-scale, high-quality RCTs that utilize ecologically valid outcome measures and extend assessments to longer-term time points to ensure comprehensive reporting (332).

Furthermore, mindfulness-based interventions (MBI) have gained recognition for their effectiveness in mitigating stress, depression, anxiety and fatigue while enhancing HQRoL and sleep quality among adults with MS (334–344). MBI are not only cost-effective (337) but they are also feasible techniques to adopt by older adults. Once the technique is learnt, it enables individuals to independently engage in the practice. However, the most optimal MBI method and the ideal frequency for achieving sustained long-term benefits continues to be unclear.

##### 2.2.2.4 Other interventions

Occupational therapy has also proved to be effective for improving the functionality of MS patients in tasks such as dressing, bathing, and ambulation (345). Fatigue management programs delivered by occupational therapists are also effective in improving MS symptoms (346), including fatigue, manual dexterity, falls prevention, cognition, depression and QoL (347). Future studies will determine whether the outcomes of occupational therapy vary in relation to the method of delivery (one-on-one versus group) and the setting of the therapy (outpatient, inpatient, or therapy administered at home) (348).

Speech and swallowing therapy is particularly important when MS has affected vocal or swallowing muscles (349, 350), which can reduce HRQoL specially in the last stages of MS (349). Electrical stimulation and botulinum toxin treatment have showed positive effects on dysphagia (351). A survey of 5,289 patients with MS in Sweden showed that 80% of participants experienced at least one speech or communication issue, including symptoms like speech fatigue or unclear articulation, while 25% indicated they faced challenges with swallowing. However, few of these patients received speech and language pathology services (352).

Most MS patients present symptoms when their body temperature is increased. Active and passive cooling methods have been used as a complimentary therapeutic approach to address symptoms sensitive to temperature (353), with no specific cooling garment identified as superior (354). The benefits of cooling therapies have been reported in physical activity and function, fatigue, and the QoL in individuals with MS (354, 355).

Limited research exists evaluating the impact of orthoses on balance in patients with MS, with studies focusing on ankle-foot and foot orthoses yielding inconclusive outcomes. One recent meta-analysis indicates that orthotic interventions appear not to enhance balance in people with MS (251).

Lifestyle modifications, including diet, may help improve MS symptoms and HRQoL (356). This can include vitamin D supplementation (357–359), probiotics (360), or Mediterranean diet (361). The evidence for the benefits of ketogenic diet and fasting in MS patients is still inconclusive (362), and ongoing studies will provide further evidence on their effects not only in MS symptomatology but also on disease progression (363).

Strong social support networks, including family, friends, support groups, or community organizations, can significantly impact the HRQoL and functioning of people with MS. MS symptoms, especially fatigue, hinder patients’ ability to form relationships and engage in social or daily activities (364). Online peer support serves as an alternative for those who cannot reach in-person groups, mitigating social isolation, yet the symptoms of MS might complicate the use of technological devices (365).

### 2.3 MS care units

As we have extensively reviewed in the prior sections, the landscape of MS treatment encompasses an array of DMTs, most of them with lack of evidence in older adults, coupled with an extensive selection of both pharmacologic and non-pharmacologic symptomatic therapies. This complexity has underscored the necessity for specialized MSCU, as the management of MS patients extend beyond the expertise of family physicians and general neurologists (18).

Central to the function of these MSCU is a multidisciplinary team, which, in addition to MS-specialized neurologists and nurses, would include neuropsychologists, clinical psychologists, physiotherapists, occupational therapists, and administrative support staff (18). In older adults with MS, a collaboration with an internal medicine specialist or geriatrician would ensure a more comprehensive approach to patient care by integrating specialized knowledge on aging-related health issues. Additionally, strong collaboration with social workers might also be key for supporting patients’ functional capacity and connection with the job market, especially as retirement ages rise across Europe. Collaboration with other specialists such as a speech therapist, dietitian, and specialists in pain, spasticity and continence is also recommended (18). Some of these services may, however, be provided by a MS neurologist or MS nurse in the MS Care Unit with special education and interest.

Moreover, the MSCU should ensure the access in a reasonable time at all the investigational procedures for diagnostic and monitoring purposes, including MRI, optical coherence tomography, evoked potentials, CSF and hematological tests. Finally, the services of neurorehabilitation are central to the management of a person with MS. This is particularly true for aged individuals in order to reinforce the mechanisms of plasticity, which efficiency decreases with age.

When the fulfillment of MSCU international criteria has been evaluated in European countries such as Hungary, half of MSCU provided sufficient care for MS patients (366). Local aspects should be considered for the implementation of MSCU worldwide. Some recommendations on the objectives, human and technical resources, and the general functioning of MSCU for specific regions have been made (367).

## 3 Advance care planning

Fear of the future has been found to be a prevailing worry among patients with MS. Some specific fears were potential further losses of mobility and independence, the prospect of becoming reliant on caregivers, and the possibility of transitioning to a nursing home (368). These insights highlight some challenges that should be addressed, such as implementing interventions that empower older adults with MS to feel more in control of their future, multidisciplinary collaboration to help families impacted by MS, and promoting community support for individuals with MS.

Advance care planning allows patients to plan their future medical care by identifying values and treatment preferences, discussing these with family and healthcare professionals, and appointing a representative to make decisions if they cannot. Discussions with neurologists about treatment restrictions have been observed to start late, usually when significant physical and cognitive decline has occurred (369).

Patients frequently imply desire to engage in conversations about death and dying (370), and it is important for healthcare professionals to recognize and foster these discussions. The European Academy of Neurology (EAN) on palliative care of people with severe progressive MS states that patients should be encouraged to discuss their wishes about future care, including the restriction of interventions and the consideration of accelerated death (371).

## 4 Final considerations

### 4.1 MS management: personalization and monitoring

The pathophysiology and immunology of MS in elderly patients, characterized by the complex interplay of the immunosenescence and inflammaging processes with neurodegeneration, highlight the need for a tailored approach to the management of MS in older adults. Taking into account the unique aspects of the disease in this population, the choice of DMT must be approached on a case-by-case basis, particularly in older patients, weighing potential risks against therapeutic benefits. Interactions with other treatments for existing comorbidities, treatment monitoring related to special conditions, the need for pharmacologic and non-pharmacologic symptomatic therapy, and the possibility of DMT discontinuation should be considered and discussed with the patient. This personalized approach underscores the importance of continuous monitoring and adjustment of the management plan to address the needs of older individuals with MS. The evaluation of biological age, in addition to chronological age, may help adapt treatment to each patient in the future. Such a complex approach requires a high level of organization that can be reached with appropriate models of assistance as the MSCU model.

### 4.2 Study design: inclusion of a wider range of age

We advocate for the inclusion of a broader age range in the design of clinical trials that evaluate the efficacy and safety of DMTs in MS. There are several reasons for this suggestion. Firstly, the demographic shift in MS prevalence towards older age groups requires a better understanding of DMT efficacy and safety in these populations. As the pathophysiology and immunology of MS evolve with age, older patients may respond differently to treatments compared to younger patients. Secondly, older MS patients often present with several comorbidities and a different pharmacokinetic profile, which can influence the efficacy and safety of DMTs. Without adequate representation of older adults in clinical trials, there is a risk of underestimating potential AEs or overestimating the efficacy of DMTs in this group. Thirdly, including a wider age range enhances the generalizability of trial results, making findings more applicable to the broader MS population. We also encourage age-specific trials and real-world studies to better understand the efficacy and safety of DMTs in the older MS population.

## Author contributions

ÓF: Conceptualization, Writing – review & editing. PS: Writing – review & editing. GC: Writing – review & editing. PV: Writing – review & editing. H-PH: Writing – review & editing. LL: Writing – review & editing. TB: Writing – review & editing. BVW: Writing – review & editing. CO-G: Conceptualization, Writing – review & editing.
